# PIP_2_ Interacts Electrostatically with MARCKS-like Protein-1 and ENaC in Renal Epithelial Cells

**DOI:** 10.3390/biology11121694

**Published:** 2022-11-24

**Authors:** Qiang Yue, Otor Al-Khalili, Auriel Moseley, Masaaki Yoshigi, Brandi Michele Wynne, Heping Ma, Douglas C. Eaton

**Affiliations:** 1 Division of Nephrology, Department of Medicine, Emory University, Atlanta, GA 30322, USA; 2 Division of Nephrology & Hypertension, Department of Internal Medicine, University of Utah, Salt Lake City, UT 84132, USA; 3 Department of Physiology, Emory University, Atlanta, GA 30322, USA

**Keywords:** MARCKS-like protein-1, PIP_2_, MARCKS-like-1, ENaC, mpkCCD cells

## Abstract

**Simple Summary:**

Epithelial Sodium Channel (ENaC) is a renal ion channel responsible for a major fraction of total body sodium balance. MARCKS-like Protein-1 (MLP-1) is a membrane protein that controls the distribution of membrane phosphatidylinositol 4, 5-*bis*phosphate (PIP_2_). PIP_2_ strongly activates ENaC with a half-activating concentration of 21 ± 1.17 μM. Normal channel activity requires MLP-1 associated with the inner leaflet of the cell membrane. MLP-1′s strongly positively charged effector domain sequesters PIP_2_ electrostatically and increases the local concentration of PIP_2_ over a hundred-fold. By controlling local PIP_2_ concentration, MLP-1 controls ENaC activity and, consequently, total body sodium balance.

**Abstract:**

We examined the interaction of a membrane-associated protein, MARCKS-like Protein-1 (MLP-1), and an ion channel, Epithelial Sodium Channel (ENaC), with the anionic lipid, phosphatidylinositol 4, 5-*bis*phosphate (PIP_2_). We found that PIP_2_ strongly activates ENaC in excised, inside-out patches with a half-activating concentration of 21 ± 1.17 µM. We have identified 2 PIP_2_ binding sites in the N-terminus of ENaC β and γ with a high concentration of basic residues. Normal channel activity requires MLP-1’s strongly positively charged effector domain to electrostatically sequester most of the membrane PIP_2_ and increase the local concentration of PIP_2_. Our previous data showed that ENaC covalently binds MLP-1 so PIP_2_ bound to MLP-1 would be near PIP_2_ binding sites on the cytosolic N terminal regions of ENaC. We have modified the charge structure of the PIP_2_ –binding domains of MLP-1 and ENaC and showed that the changes affect membrane localization and ENaC activity in a way consistent with electrostatic theory.

## 1. Introduction

**Figure 1:**
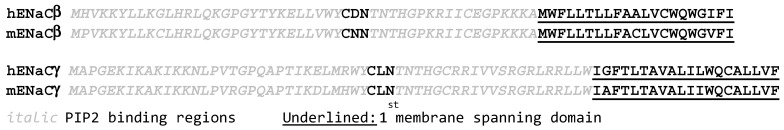
Sequences of the N-terminal regions of human and mouse β and γ ENaC with PIP2 binding domains and transmembrane-spanning domains marked.

Twenty years ago, cytosolic phosphatidylinositol-4,5-*bis*phosphate (PIP_2_) was shown to increase the open probability of the epithelial sodium channel (ENaC) [1]. Since then, we and several others have also shown that ENaC P_o_ is increased by cytosolic PIP_2_ [1,2,3,4,5,6]. The implication of these electrophysiological experiments is that PIP_2_ must interact with one or more of the ENaC subunits. We and others have shown that PIP_2_ interacts with ENaC β and γ, but not α subunits, at two binding sites for PIP_2_ at the cytosolic N-terminus of ENaC β and γ [2,4,7,8,9] (Figure 1). Other sites for PIP_2_ binding have been described [3,4,10,11,12,13], but the N-terminal sites appear to be the critical sites for PIP_2_ regulation of ENaC. Several reports have shown that the positive charges within the amino terminus of β- and γ-ENaC interacts with the anionic phospholipids like PIP_2_ in the inner leaflet of the plasma membrane to regulate ENaC activity [4,12,14,15]. We know that PIP_2_ is required for normal ENaC activity since when phospholipase C is activated to hydrolyze PIP_2_, it leaves no PIP_2_ available for binding to ENaC, and ENaC Po falls close to zero [13,14,15]. This implies that the Po of ENaC without a PIP_2_ bound is zero or close to zero and that PIP_2_ is necessary to open ENaC. 

### MLP-1 Associates with PIP2

The observations described above seem to imply that PIP_2_ associates with ENaC, and when it does, PIP_2_ causes ENaC to enter an open state. When PIP_2_ disassociates from ENaC, the channel closes. This means that if we knew the concentration of PIP_2_ and ENaC in the membrane and their diffusion constants, we could predict the opening rate of the channel. 

There are only about seven functional ENaC per µ^2^ in the apical membrane of principal renal cells [16], and PIP_2_ constitutes less than 1 in 1000 membrane lipid molecules [17]. The diffusion constant of PIP_2_ measured by fluorescent correlation spectroscopy is about 4 × 10^−12^m^2^/s [18]. If ENaC diffusion is slow and if PIP_2_ follows a random walk, then PIP_2_ and ENaC should only interact on average every 6.3 × 10^2^ s (about once in 10 min). However, the observed rate of ENaC opening in principal cells is every one or two seconds in a typical patch [19]. Therefore, we suggest that MARCKS-like Protein 1 (MLP-1) is a membrane protein that binds both ENaC [1] and PIP_2_ [20] so that the PIP_2_ can activate nearby ENaC to account for the anonymously high opening rate. MLP-1 contains a highly positively charged domain that will electrostatically bind PIP_2_ [21,22,23,24,25,26]. 

## 2. Methods

### 2.1. Tissue Culture Models

**Figure 2:**
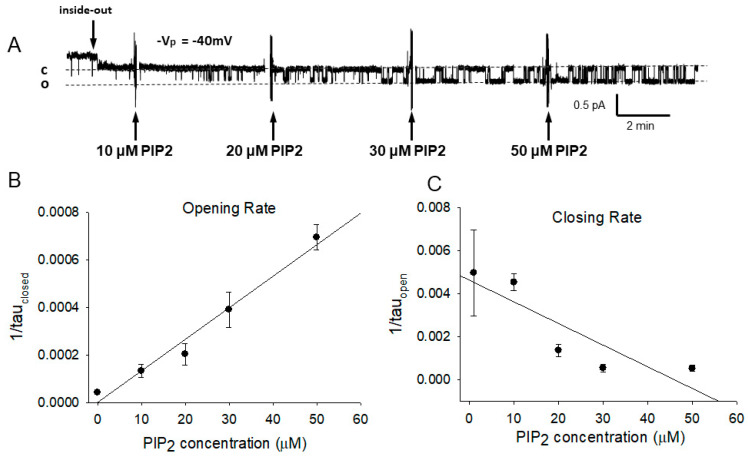
PIP_2_ activates ENaC. (**A**). Single channel record from an excised cell-free patch from an H441 epithelial cell. After excising the patch bathed in a solution that mimics the intracellular ion composition, we added increasing doses of PIP_2_ to the cytosolic surface of the patch. Mean closed time decreases. This implies an increase in the opening rate as shown in Panel (**B**) (slope = 7.97 × 10^−6^ ± 1.63 × 10^−6^ ms^−1^ × mM^−1^ *p* = 0.0165). There was also a smaller increase in open time (Pane (**C**)), implying a decrease in closing rate, although the slope may not be significant (slope = −1.01 × 10^−4^ ± 3.27 × 10^−5^ ms^−1^ × mM^−1^ *p* = 0.0539). Data is from 11 patches with 56, 1076, 418, 233, 327 events at 0, 10, 20, 30, and 50 mM PIP_2_ concentrations, respectively.

We will continue to use several tissue-culture models. mpkCCD cells are derived from mouse principal collecting duct cells [27]. Since they are aldosterone-responsive, have high transepithelial resistance and trans-epithelial current sensitive to amiloride, form stable monolayers, are relatively easy to transfect, have endogenous ENaC channels in patch clamp experiments, and have high levels of endogenous MLP-1, they are a useful model for examining native ENaC-PIP_2_-MLP-1 interactions. mDCT-15 cells are another mouse renal cell line originally derived from the distal convoluted tubule [28]. The cells form low-resistance confluent monolayers, have amiloride-sensitive transepithelial current, express ENaC, have low levels of MLP-1, are easy to transfect and are easy to use for single-channel measurements. We have used the cells to examine the properties of mutant MLP-1 and their effect on ENaC regulation [1]. We have cellular MLP-1 knockouts for both mpkCCD and mDCT-15 cells which will be critical for examining the role of MLP-1 in ENaC and PIP_2_ interactions. H441 cells are lung-derived epithelial cells that have endogenous MLP-1, can be easily transfected and robustly express ENaC channels. The cells are relatively easy to form inside-out, cell-free patches that can be used to study the effects of exogenous PIP_2_ on ENaC (Figure 2). 

### 2.2. Fluorescent Immunohistochemistry

Fluorescent immunohistochemistry was performed on monolayers of live epithelial cells. Briefly, cells were treated with anti-γ-ENaC (StressMarq, Inc., Toronto, CA, Canada) in a serum-containing medium and detected with an antirabbit GFP secondary antibody (Sigma, Inc., St. Louis, MO, USA). Subsequently, cells were treated with cholera toxin B (CTxB) subunit conjugated with AlexaFluor 488 and then treated with anti-CTxB antibody (both from Molecular Probes, Inc., Eugene, OR, USA). For some monolayers, cells were treated with PBS followed by 2% paraformaldehyde and then with 0.1% triton ×100 in PBS before treating with anti-ENaC antibodies and AlexaFluor 488 secondaries. 

For live cell imaging, mDCT-15 or mpkCCD cells were seeded on 24 mm transwell permeable supports (Corning) in growth media (50:50 DMEM:F12, Gibco Thermo Fisher, Waltham, MA, USA). A Leica SP8M two-photon microscope with a 25× NA 0.95 lens was used to examine the fluorescence and capture images of *xyz*-stacks as previously described [1].

### 2.3. Data Analysis and Statistics

All data acquisition and analysis were performed as described previously [29,30]. Data are reported as means ± se. Statistical analysis was performed with SigmaPlot software (version 14.0, Impixon, Inc., Palo Alto, CA, USA). Differences between groups were evaluated with one-way ANOVA. Results were considered significant if *p* < 0.05. 

All data were presented as means ± SD and the number of samples (n). One-way ANOVA or Kruskal–Wallis one-way analysis of variance on ranks was used to compare multiple groups with Holm–Sidak or Dunn’s post-tests. The *p*-value of <0.05 was considered statistically significant. 

### 2.4. Co-Localization Analysis

To determine if ENaC subunits colocalize with lipid raft markers, monolayers were stained with rabbit primary antibodies to α, β, or γ ENaC and a lipid raft marker., CTX-B. Following treatment with fluorescent secondary antibodies, ENaC (green) and raft marker (red) were examined using confocal microscopy on an Olympus Fluoview 1000 confocal microscope 20× (NA 0.75) or 40× (NA 1.3). To determine co-localization, we first determined that there was no fluorescence bleed-through from the green channel to the red channel or vice versa. Following treatment with fluorescent secondary antibodies, ENaC (green) and raft marker (red) were examined using confocal microscopy on an Olympus Fluoview 1000 confocal microscope 20× (NA 0.75) or 40× (NA 1.3). To determine co-localization, we first determined that there was no fluorescence bleed-through from the green channel to the red channel or vice versa. Then we merged images from the green channel and the red channel; yellow in the merged image was an indication of co-localization. To produce a more quantitative measure of co-localization, we used the co-localization algorithms in the image analysis program ImageJ (version 1.53u) [31,32,33]. These algorithms examine the merged images pixel by pixel for red and green intensities and establish thresholds for intensity below which there is no significant correlation between red and green. 

This represents an unbiased method for determining thresholds. Pixels that have both red and green intensities above the thresholds are analyzed and re-colored white. In addition, the number and fraction of pixels with red-green co-localization is calculated, and red-green and green-red correlation coefficients are calculated (Manders coefficients, *m*_1_ and *m*_2_) according to Equation (1):

(1) $$\begin{array}{cc} m_{1}=\frac{\sum_{i}s1_{i}\cdot s2_{i}}{\sum_{i}{\left(s1_{i}\right)}^{2}} & m_{2}=\frac{\sum_{i}s1_{i\cdot }s2_{i}}{\sum_{i}{\left(s2_{i}\right)}^{2}} \end{array}\;\;\;$$

where *s*1*_i_* and *s*2*_i_* are the red and green intensities of the *i*^th^ pixel in the image. Only pixels with significant intensities in both channels will contribute to the coefficient.

### 2.5. Western Blots

We performed the Western blots and densitometric analysis using methods and antibodies that we have previously reported [1]. Membranes were imaged with the Odyssey imaging system (Licor Biosciences). Anti-MLP-1 antibodies detect the same molecular weight bands as anti-epitope antibodies for V5, 3XFLAG, and mCherry fusion proteins with MLP-1. Specific antibodies are described in the supplemental material.

### 2.6. Single-Channel Patch-Clamp Studies

Basic single-channel methods have been previously described [1]. Single channel events were recorded with an Axopatch 1D and then digitized at 4000 Hz with an Axon 1440 digitizer directly to disk storage.

Patch pipettes were fabricated from filamented borosilicate glass capillaries (TW-150F; World Precision Instruments) with a two-stage vertical puller (PP-2; Narishige, Tokyo, Japan) with a resistance of 6–10 MΩ. mpkCCD cells treated with 1 μM aldosterone and cultured on 24-mm permeable Transwell supports (Corning). Cells were visualized with Hoffman modulation optics (Modulation Optics, New Haven, CT, USA) on a Nikon Diaphot. Negative pressure was applied to achieve a cell-attached patch with a seal resistance of 10–20 GΩ after making contact between the pipette tip and the cell surface. The extracellular bath solution consisted of a saline solution (150 mM NaCl, 5 mM KCl, 1 mM CaCl_2_, 2 mM MgCl_2_, 5 mM glucose, and 10 mM HEPES, adjusted to pH 7.4). The patch pipette solution consisted of a saline solution (140 mM LiCl, 2 mM MgCl_2_, and 10 mM HEPES, adjusted to pH 7.4). The cell-attached patch configuration was used for single-channel experiments, and voltages are given as the negative of the patch pipette potential. All single-channel events were recorded with an Axopatch 1D and then digitized at 1000 Hz with an Axon 1420 digitizer directly to disk storage.

Otherwise, all experiments used cell-free, inside-out patches. Initially, both the apical side and basolateral side of the monolayer were bathed in the same saline. Once a seal was formed, the apical bath solution was exchanged for saline that mimics the intracellular ion environment (in mM): 3 NaCl, 140 KCl, 4 CaCl_2_, 1 MgCl_2_, 10 HEPES, and 5 EGTA (yields 100 nM free Ca^2+^) at pH 7.4 (titrated with 1 N KOH). After baseline recording from the excised patch, phosphatidylinositol-4,5-*bis*phosphate from the bovine brain (Avanti Polar Lipids, Birmingham, AL, USA) was added to the cytosolic surface of the patch. Experiments were performed at room temperature (22–23 °C). 

ENaC activity and open and closed times within a patch were calculated using pCLAMP 10 software (version 10.7, Molecular Devices, San Jose, CA, USA). We used the product of the number of channels (*N*) times the single channel open probability (*P_o_*) as a measure of channel activity within a patch. This product was calculated without making any assumptions about the total numbers of channels in a patch or the P_o_ of a single channel:

(2) $$NP_{o}=\overset{N}{\underset{n=1}{\sum }}\frac{{\mathrm{nt}}_{n}}{T}$$

where T is the total recording time, n is the number of channels opening, and t_n_ is the open time for the n channels. The total number of functional channels (*N*) in a patch was estimated by observing the number of peaks in the current-amplitude histogram over the entire duration of the recording, after which *P_o_* could be calculated from *NP_o_* and *N*.

### 2.7. Chemicals

Most chemicals were obtained from Sigma-Aldrich (St. Louis, MO, USA), including methyl-β-cyclodextrin and lanthanum chloride. Phosphatidylinositol-4,5-*bis*phosphate from the bovine brain was obtained from Avanti Polar Lipids (Birmingham, AL, USA). 

## 3. Results

### 3.1. PIP_2_ Activates ENaC

There are previous reports by us and others that PIP_2_ activates ENaC in excised, inside-out, cell-free patches [2,3,4,5,6,7]. Ma et al. found that PIP_2_ activation of ENaC resulted from a change in ENaC conformation that leads to an increase in channel open probability. We examined this question further by examining the amount of PIP_2_ necessary to activate ENaC and the kinetics of the activation (Figure 2). As expected, the addition of PIP_2_ increases ENaC activity specifically by significantly decreasing the mean closed time of single-channel events (there is also an apparent increase in mean open time that is only marginally significant). Using methods to pharmacologically alter PIP_2_ in renal epithelial cell membranes, Pochynyuk et al. also observed a decrease in closed times and an increase in open times for single ENaC channels [14]. We fit the open probability vs. PIP_2_ concentration to the Hill equation:

(3) $$P_{o}=\frac{P_{max}}{1+\frac{K_{0.5}}{{\left[PIP_{2}\right]}^{n}}}\;\;\;\;\;\;\;\;$$

**Figure 3:**
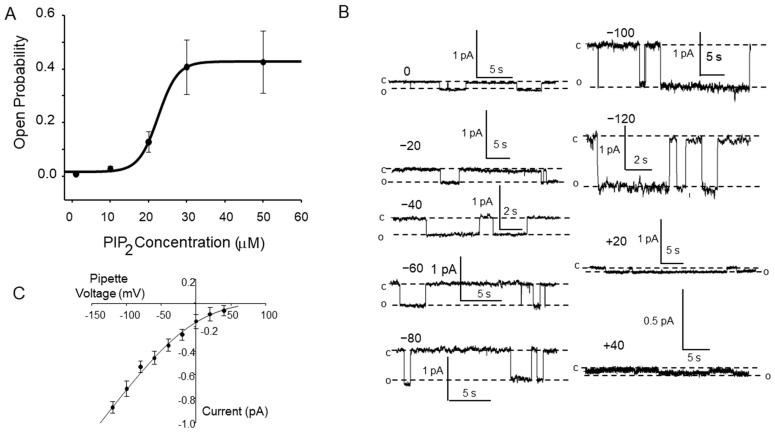
Dose-response relationship for PIP_2_ activation of ENaC and current-voltage relationship for ENaC in excised patches from H441 cells. In Panel (**A**), we fit the open probability vs. PIP_2_ concentration to the Hill equation (see text). The half-activating concentration of PIP_2_ was 22 ± 0.66 μM with a maximal open probability of 0.411 ± 0.0156. The Hill coefficient is 2.5 ± 0.55 (r^2^ = 0.949). In Panel (**B**), we show representative single-channel events at ten different voltages. The vertical scale bars are the same in all records except +40. The horizontal scale bars vary among records. Channel openings (inward current) are downward. Open and closed levels are indicated with dashed lines labeled “o” and “c,” respectively. Panel (**C**) shows the single-channel current-voltage relationship of 4l channels from excised patches. We fit the single channel current to the Goldman Current Equation [34] that gave a unit conductance of 6.6 ± 1.4 pS between −120 and −40 mV. It also predicted the observed inward rectification at positive potentials and a reversal potential of 83 ± 3.1 mV. These numbers are consistent with ENaC exposed to high 140 mM LiCl in the pipette and low 3 mM Na^+^ on the cytosolic surface.

The half-activating concentration of PIP_2_ was 22 ± 0.66 μM with a maximal open probability of 0.411 ± 0.0156. The Hill coefficient is 2.5 ± 0.55 (Figure 3A). We also measured the single-channel current-voltage relationship of several channels in excised patches (Figure 3B). We fit the single channel current to the Goldman Current Equation [34] that gave a unit conductance of 6.6 ± 1.4 pS between −120 and −40 mV. It also predicted the observed inward rectification at positive potentials and a reversal potential of 83 ± 3.1 mV. These are the characteristics we would expect for ENaC with 140 mM Na^+^ on the apical surface and 3 mM Na^+^ on the cytosolic surface.

### 3.2. PIP_2_’s Interaction with ENaC Is Consistent with an Electrostatic Interaction

**Figure 4:**
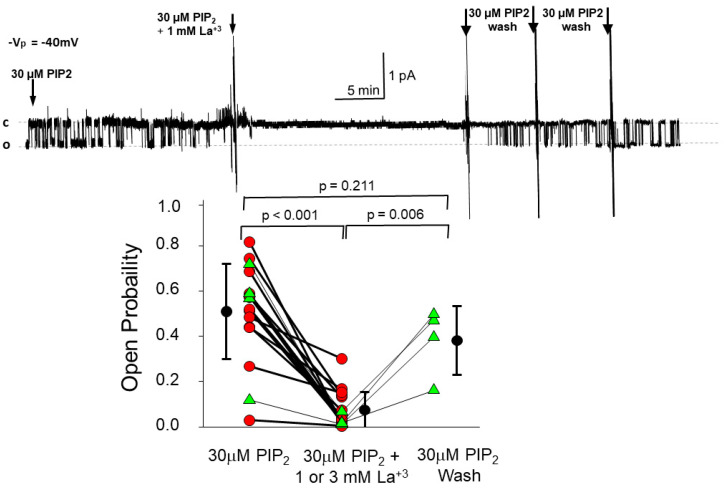
Lanthanum, a trivalent cation, reduces PIP_2_ activity. We excised patches from H441 cells and added 30 μM PIP_2_ in PBS to the cytosolic surface to activate ENaC. After 12 min, we added 1 mM La^3+^Cl_3_^−^ which rapidly reduced PIP_2_-induced ENaC activity. In four experiments, we washed away the La^3+^ with PIP_2_ -containing PBS, which rapidly reversed the reduction in ENaC activity. The P_o_ of the channel before adding lanthanum is significantly different from after lanthanum (*p* < 0.001) but is not different than after wash out (*p* = 0.211); however, wash out is also significantly different from the lanthanum treated patch (*p* = 0.006). (by repeated measures ANOVA).

McLaughlin and his coworkers have suggested that membrane or membrane-associated proteins that have clusters of positive charge likely interact with PIP_2_ by electrostatic interaction rather than by more traditional ligand binding. The N-terminal PIP_2_ binding sites on ENaC are strongly positively charged (Figure 1), suggesting that PIP_2_ interaction with ENaC is likely an electrostatic interaction between the strongly negatively charged lipid PIP_2_ with the positively charged regions of ENaC. Electrostatic interactions can be distinguished from ligand-mediated covalent interactions by the ability to screen the charge transfer between PIP_2_ and ENaC with a mobile polyvalent ion. Zhang et al. cells [12] found that polycationic polylysine could block PIP_2_ -induced ENaC activity in excised patches from renal epithelial cells. In support of this observation, we used trivalent cationic lanthanum to screen PIP_2_’s negative charge. We used 30 mM PIP_2_ to increase ENaC activity in an excised patch. The addition of 3 mM La^3+^ strongly reduced PIP2 activity (Figure 4). In a few experiments, we showed that the blocking effects of lanthanum were rapidly reversible. Blocking PIP_2_-induced ENaC activity with La^3+^ and poly-cationic lysine show that PIP_2_/ENaC interaction could be electrostatic, but other explanations are also possible. Others have suggested that PIP_2_-ENaC interactions are likely electrostatic [35,36].

### 3.3. ENaC Is in Lipid Domains Enriched in Inositol Lipid Phosphates

**Figure 5:**
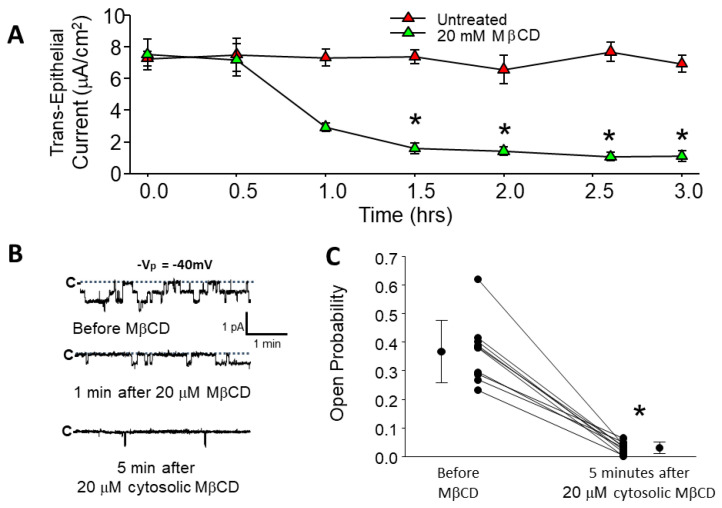
Sensitivity to methyl-β-cyclodextrin (MβCD) is asymmetric. (**A**) To inhibit sodium transport in cells in monolayer cultures on permeable supports required treatment with high concentrations (20 mM) of MβCD on the apical surface. * indicates significant difference from untreated. (**B**) In contrast, low concentrations of MβCD on the cytosolic surface of an excised, cell-free patch treated with 20 μM PIP_2_ rapidly reduces ENaC P_o_ in excised, cell-free patches. We excised membrane patches from mpkCCD cells in the presence of 20 μM PIP_2_ and applied 20 μM MβCD to the cytosolic surface of the membrane, where it presumably would have direct access to the cholesterol in the inner leaflet of lipid rafts. (**C**) At a concentration 1000 times lower than what we had applied to the luminal surface (20 μM instead of 20 mM), we could reduce ENaC open probability close to zero in less than 5 min (*p* < 0.001, paired *t*-test n = 7 where symbols represent individual experiments).

**Figure 6:**
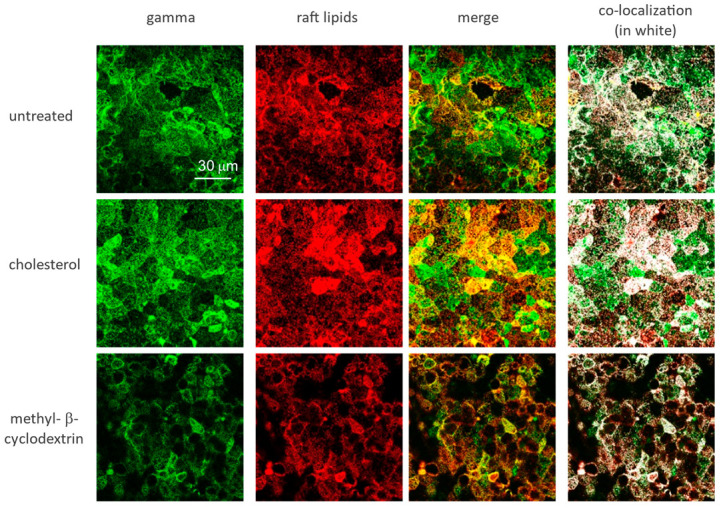
Raft lipids and γ-ENaC density are correlated with cholesterol in the membrane. We show images from confluent monolayers imaged with a water immersion 25× lens on an Olympus Fluoview confocal microscope (scale bar 30 μm). Cells were either untreated, cholesterol treated (30 μg/mL), or treated with methyl-β-cyclodextrin (MβCD) (20 mM). We measured the mean intensity and standard deviation of intensity of all pixels in each image and tested these for significant differences. For γ-ENaC, the untreated and cholesterol-treated cell intensities are significantly different from the MbCD-treated cells (*p* = 0.014 and <0.001, respectively). Untreated and cholesterol-treated intensities are not different (*p* = 0.258). The same is true for the intensities of raft lipids: MβCD intensity is less than untreated and cholesterol-treated (*p* = 0.005 and <0.001 for both), and untreated and cholesterol-treated are not different (*p* = 0.385). For the merged images and the co-localization, the intensities of all images are significantly different from one another: untreated and cholesterol-treated vs. MβCD-treated at the *p* < 0.001 level and untreated intensity are less than cholesterol-treated (*p* = 0.01 for merged and *p* = 0.025 for co-localization intensities). For the right panels, we used the “colocalization finder” plugin of “Image J” (8) to analyze our images for colocalization of the labels for CTX-B and γ-ENaC antibodies. This plugin allows the user to identify pixels that contain both channel 1 (γ-ENaC fluorescence–green) and channel 2 (CTX-B fluorescence–red). These pixels are then highlighted in white. We restricted the analysis to pixels having ratios of intensity values in the two channels greater than 0.1.

**Figure 7:**
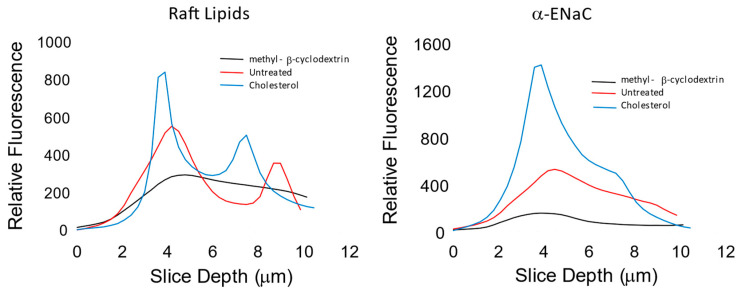
Cholesterol alters the distribution of α-ENaC and raft lipids. We used the z-axis scanning capability of a Leica SP-8MP two-photon microscope to measure the density and relative location with respect to the apical membrane of raft lipids and α-ENaC after no treatment, the addition of exogenous cholesterol, and after 5 min of basolateral MβCD (20 mM) (as in Figure 6). Raft lipids were detected with the cholera toxin B subunit. Slices were made at 0.1 μM intervals. There are raft lipids in both apical and basolateral membranes, but more lipids in the apical membrane than in the basolateral membrane. After exogenous cholesterol (30 μM/mL), raft lipids increase above those in untreated cells; and decrease after MβCD. α-ENaC follows the same pattern at the apical membrane; we speculate that the different height of the cells likely has to do with changes in sodium transport activity associated with changes in functional ENaC.

**Figure 8:**
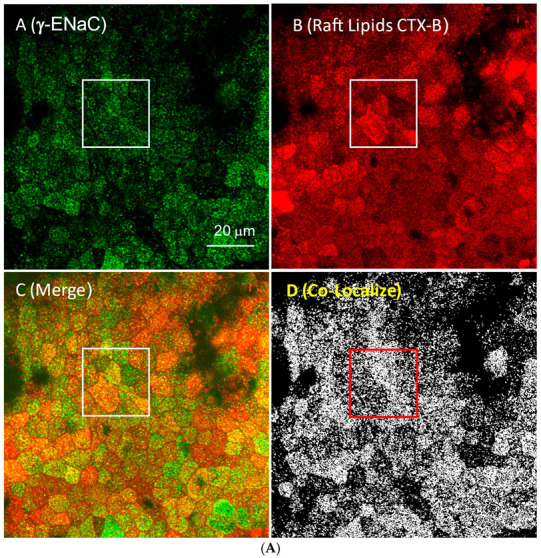

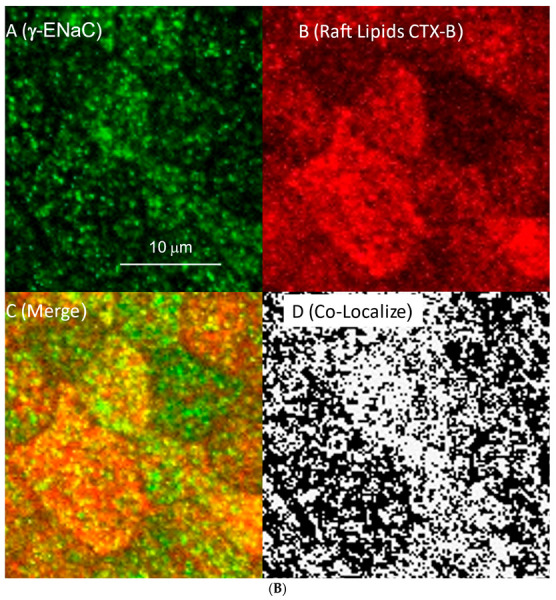
(**A**) γ-ENaC co-localizes with the lipid domain marker cholera toxin-B. The first image in the upper left shows the apical surface of mpkCCD cells labeled with an anti-γ ENaC antibody, while the upper right shows the CTX-B labeling corresponding to glycosphingolipids in lipid domains. The lower left shows the merged images with yellow-orange indicating co-localization. Panel D shows in white the locations in the upper panels where a red pixel and a green pixel of similar intensities overlap. The co-localization coefficient is 0.783. There are areas that are green with little red, but few areas of red that do not colocalize with green implying that specialized lipid domains have ENaC. We used the “colocalization finder” plugin of “Image J” (8) to analyze our images for colocalization of the labels for CTX-B and γ-ENaC antibodies, as we did in Figure 6. This plugin allows the user to identify pixels that contain both channel 1 (γ-ENaC fluorescence–green) and channel 2 (CTX-B fluorescence–red). These pixels are then highlighted in white on separate images. We restricted the analysis to pixels having ratios of intensity values in the two channels greater than 0.1. (**B**) The images below are magnified images of the inset areas of the larger image to better visualize cells and colocalization.

We and others have proposed in previous work that functional ENaC is confined to specific membrane domains [37,38], often referred to as lipid rafts that contain high concentrations of cholesterol, inositol phospholipids, and the enzymes which produce the lipids [39,40,41]. Others have shown that depletion of cholesterol with methyl-β-cyclodextrin (MβCD) or the cholesterol synthesis blocker, lovastatin, disrupts the rafts and the addition of exogenous cholesterol increases raft area [42,43,44]. The same work also showed that increasing or decreasing raft area by altering cholesterol increased or decreased membrane PIP_2_ and increased or decreased ENaC subunit density in the membrane. In addition, the work also showed that there was a strong asymmetry in the access of MβCD to cholesterol; it takes approximately 10 times longer and 1000 times more MβCD to reduce ENaC activity and presumably remove cholesterol from the outer leaflet vs. the inner leaflet (Figure 5). We confirmed these results in additional ways. We show qualitatively, using confocal methods, that γ-ENaC density correlates with the density of raft lipids and with increasing or decreasing cholesterol (Figure 6). Next, we used the z-axis scanning capability of a two-photon microscope to measure the relative location with respect to the apical membrane of raft lipids and α-ENaC with the addition of exogenous cholesterol, no treatment, and after 5 min of basolateral MβCD (Figure 7). As might be expected, there are raft lipids in both apical and basolateral membranes, but more apical lipids after exogenous cholesterol, somewhat less in untreated cells, and the least after MβCD. α-ENaC follows the same pattern at the apical membrane; we speculate that the different height of the cells likely has to do with changes in sodium transport activity associated with changes in functional ENaC. Finally, we repeated the original experiment at a higher resolution to better show the co-localization of γ-ENaC with raft lipids (Figure 8A,B) which show γ-ENaC present in lipid domains (orange in the merged image) and non-domain regions (green in merged) of the apical membranes of renal cells. In Table 1, we quantify the ENaC and raft lipid co-localization using the colocalization threshold plugin of the FIJI image analysis program. The Table shows that the colocalization for all three ENaC subunits is similar, which is not surprising since ENaC in the membrane is a trimeric heteromer.

Other investigators, using differential detergent solubility, have also shown that a more than 50% of ENaC is in PIP_2_-rich domains [37,38,41,45,46,47,48]. This localization is supported by the observation that disrupting raft domains by removing or reducing membrane cholesterol with methyl-β-cyclodextrin (MβCD) or lovastatin reduces or eliminates ENaC activity [43,49]. This shows that the only functional channels are in these domains.

We hypothesize that MLP-1 acts to maintain PIP_2_ in lipid domains and promote ENaC-PIP_2_ interaction. MLP-1 associates with the inner leaflet of PIP_2_-rich lipid domains through electrostatic interactions of its basic effector domain [50], causing MLP-1 to associate with PIP_2_-rich lipid domains [20,22,51,52,53]. Published data implicate MLP-1 in increasing the local concentration of PIP_2_ near ENaC in lipid rafts leading to the anomalously high open rate of ENaC (compared to that expected from simple lateral diffusion of ENaC and PIP_2_) [20,25,26,53,54,55,56]. 

### 3.4. MLP-1 Is Necessary for ENaC-PIP_2_ Interaction

We suggest that normal channel activity requires PIP_2_ binding to MLP-1. MLP-1’s strongly positively charged effector domain sequesters PIP_2_ electrostatically [20]. Our recent data shows that ENaC covalently binds MLP-1 [1], so MLP-1-induced increases in PIP_2_ concentration would be near PIP_2_ binding sites on the cytosolic N terminal regions of β and γ ENaC. This binding is presumably also electrostatic since the ENaC binding sites contain multiple basic residues [2,4,36,57] (Figure 1). The advantage of electrostatic interaction is that PIP_2_ can remain near both ENaC and MLP-1 [20] in effect, dividing PIP_2_‘s time between the two charged domains.

**Figure 9:**
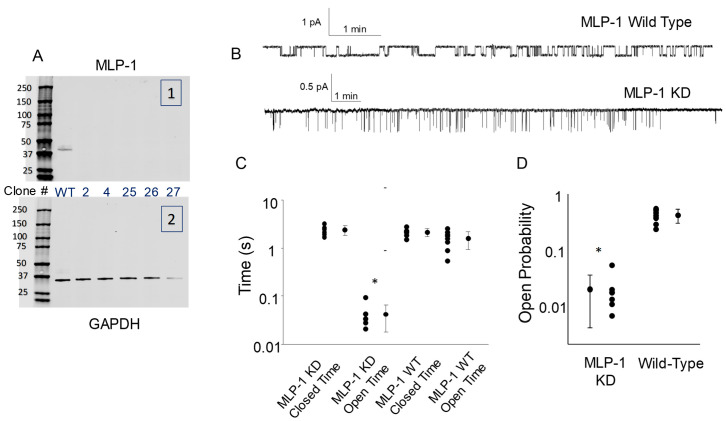
ENaC activity is greatly reduced in mpkCCD cells in which MLP-1 is strongly knocked down. Using CRISPR/Cas9, MLP-1 was knocked down between five and seven cycles (by Q PCR), implying at least a 30 to 120-fold reduction in MLP-1 in several clones. Western blots of wild-type and five knockout clones are shown in (**A**) blotted for MLP-1 (in 1) or GAPDH (in 2). MLP-1 is below detection limits. Full Western blot images are presented in supplementary data. In (**B**), single channel traces show that only short, flickery channel events are observed in the KO cells (lower trace) compared to the wild type (upper trace). Many of the events in the knockdown trace are so short that they do not reach full amplitude due to filtering (at 100 Hz). (**C**) shows that the major effect of the knockdown is to reduce the mean open time in the KD cells. The mean open time is significantly different from all other mean times (* indicates significant difference *p* < 0.05). As expected in Panel (**D**), the reduced mean open time leads to a dramatically reduced open probability (* indicates significant difference *p* < 0.001). Symbols represent values for individual experiments.

A major problem with the tissue culture cell experiments in which exogenous MLP-1 is transfected into cells is that there is endogenous MLP-1 in the cells which contributes to PIP_2_ activation of ENaC. Therefore, we cannot tell whether the low level of ENaC activity associated with an untreated inside-out, excised patches is the rate of ENaC finding free PIP_2_ (rather than that associated with MLP-1) or if it is due to endogenous MLP-1 sequestering some PIP_2_. Therefore, with the help of colleagues at the University of Utah, we used CRISPR/Cas9 to knock out MLP-1 mpkCCD cells. After several rounds of dilution cloning, we tested 40 clones by PCR for MLP-1. We found 18 mpkCCD and 24 DCT-15 clones with no detectable MLP-1. Further, analysis by qPCR found four clones with more than a five-cycle difference in expression compared to the wild-type. On Western blots, there was no detectable MLP-1 (Figure 9A). 

Single channel recordings from the knock-out cells contain only short, flickery channel openings in the KO cells compared to wild-type (Figure 9B). Many of the events in the KO trace are so short that they do not reach full amplitude due to filtering. The major effect of the knockdown is to reduce the mean open time in the KO cells. The mean open time in the KO cells is significantly different from all other mean times, both closed or open (*p* < 0.05; Figure 9C). Of course, the reduced mean open time leads to a dramatically reduced open probability (*p* < 0.001; Figure 9D). 

### 3.5. Altering the Charge Structure of β or γ ENaC Changes ENaC Open Probability

Our results so far suggest that PIP_2_ is necessary to open ENaC and that PIP_2_ interacts with ENaC via electrostatic interactions with PIP_2_ binding domains in the N-terminus of β and γ. If this is true, then altering the charge structure of the binding domains should alter the strength of the PIP_2_ interaction.

**Figure 10:**
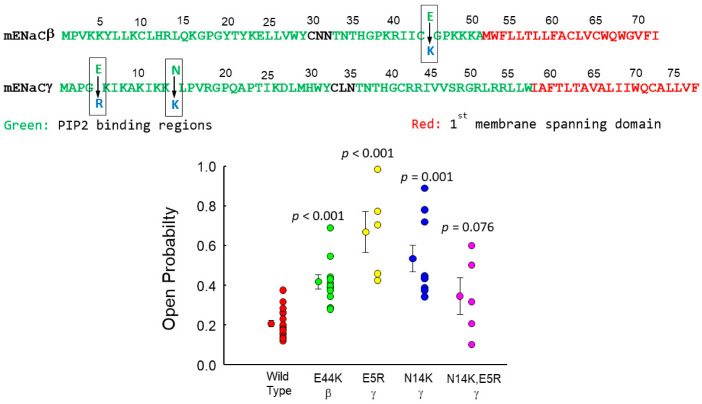
Altering the charge structure of β or γ ENaC PIP_2_-binding domains changes ENaC open probability. We made four mutations that increased the positive charge of PIP_2_ binding domains in β or γ ENaC: β E45K, γ E5R, γ N14K, and γ N14K, E5R. There is negatively charged glutamate in the middle of the sequence of cationic residues at position E45, which we have mutated to a positively charged lysine. For γ, we made one single mutation, E5R, a negative charge to a positive charge, and a double mutation, γ N14K, E5R. We transfected H441 with α-ENaC, one of the mutant ENaCs, and the other wild-type ENaC subunits. After transfection, we formed excised patches and applied 30 μM PIP2 to the cytosolic surface of the patch and examined the open probability of the patches. All four mutants increased ENaC open probability above that of wild-type subunits, but only the β E45K, γ E5R, and the γ N14K mutants increased the open probability significantly from wild-type. Wildtype Po is 0.236 ± 0.0744 (n = 11); β E45K is 0.418 ± 0.116 (n = 11, *p* = 0,014); γ E5R is 0.644 ± 0.259 (n = 5, *p* < 0.001); and γ N14K is 0.535 ± 0.204 (n = 9, *p* < 0.001) and γ N14K E5R 0.346 ± 0.205 (n = 5; *p* = 0.176). All compared to wild-type. Symbols represent individual experiments; symbols with “whiskers” are means ± s.d.

An examination of the β and γ N-terminal sequences suggests several possibilities for significant alterations in charge. For β, there is negatively charged glutamate in the middle of the sequence of cationic residues at position E45, which we have mutated to a positively charged lysine. (Figure 10). For γ, we made two single mutations, E5R and N14K and a double mutation, N14K, E5R. We transfected H441 with α-ENaC, one of the mutant ENaCs, and the other wild-type ENaC subunit. After transfection, we formed excised patches and applied 30 µM PIP_2_ to the cytosolic surface of the patch and examined the open probability of the patches. All three mutants increased ENaC open probability: but only the β E45K, the γ N14K and the γ E5R mutants increased the open probability significantly. If the association of ENaC depends upon the amount of positive charge, then the increase in activity as acidic residues are replaced with basic residues is not completely surprising. Since E5R has the largest increase in activity and N14K has the second largest increase in activity, it seems surprising that, when expressed together, the activity of the two combined mutations is less than either of the single mutations alone. 

**Table 1:** Co-localization parameters for ENaC and raft lipids.

	αENaC	βENaC	γENaC
Pearson’s correlation coefficient	0.194	0.198	0.190
ENaC channel (green) threshold	61	66	64
Raft lipids channel (red) threshold	33	31	32
Overlap coefficient (*R—pixels above threshold)*	0.761	0.731	0.802
Overlap coefficient (*R—pixels below threshold)*	0.0030	0.0029	0.0029
ENaC channel overlap coefficient (*k*_1_)	0.976	0.944	0.952
Raft lipids channel overlap coefficient (*k*_2_)	0.999	0.960	0.972
Slope of the correlation function	0.685	0.673	0.706
Intercept of the correlation function	−9	−8	−9
ENaC channel threshold	61	66	64
Raft lipids channel threshold	33	31	32
Number of colocalized pixels (*red and green > threshold*)	19,366	18,362	20,041
Total green pixels above the threshold	128,935	115,122	136,799
Total red pixels above the threshold	138,626	151,502	100,005

Values were determined according to the formulas described in Methods for the images in Figure 4 using the colocalization threshold plugin of the FIJI image analysis program.

## 4. Discussion

Figure 2 and show that phosphatidylinositol 4, 5-*bis*phosphate (PIP_2_) applied to the cytosolic surface of mpkCCD cell membranes in excised, inside-out patches activates ENaC in a dose-dependent manner by changing channel open probability. Our electrophysiological experiments show that PIP_2_ must interact with one or more of the ENaC subunits. Anti-PIP_2_ antibody co-immunoprecipitates ENaC β and γ, but not α subunits [1]. Two binding sites for PIP_2_ have been identified at the N-terminus of ENaC β and γ [11,35]. Other investigators have described additional sites for PIP_2_ and PIP_3_ binding [4,5,8,36,58], but the N-terminal sites appear to be the critical sites for PIP_2_ regulation of ENaC. 

### MLP-1 Promotes ENaC-PIP_2_ Interaction

PIP_2_ opens ENaC. MLP-1 has a crucial role in the PIP_2_ regulation of ENaC. MLP-1 binds PIP_2_ electrostatically without any direct covalent binding [20]. This means MLP-1/PIP_2_ association contrasts with the binding of a more typical ligand to a specific receptor. First, the MLP-1 charge acts at a distance. The coulombic force exerted by MLP-1 on PIP_2_ decreases as the square of the distance between the MLP-1’s positive charge domain and PIP_2_, but the distances within the membrane are relatively small, and the membrane dielectric constant is also very small. With no MLP-1, PIP_2_ moves within the membrane lipid in a two-dimensional random walk, but MLP-1’s positive charges biases the movement, so PIP_2_ always moves towards MLP-1. This means that MLP-1 increases the local concentration of PIP_2_ [25,26,51]. MLP-1’s membrane concentration is about 10 μM, which is similar to the concentration of PIP_2_ [51]. Our prior work shows that the N-terminal domains of β and γ ENaC bind the MH-2 domain of MLP-1 [1]; therefore, the increased concentration of PIP_2_ would be in close proximity to PIP_2_ binding sites on the cytosolic N terminal regions of ENaC. ENaC binding is also electrostatic since the binding sites contain multiple positive residues [2,4,36,57]. The advantage of electrostatic interaction is that PIP_2_ can simultaneously be near both ENaC and MLP-1 [20].

Besides the simple coulombic forces, MLP-1 has other properties that enhance PIP_2_ interaction. First, MLP-1’s positive charge is dragged below the surface of the membrane by hydrophobic phenylalanines, which contribute approximately 4 Kcal/mol of hydrophobic interaction energy each [20] to force the positive charge into the head group region of the membrane lipid phase. The lipid phase is a much lower dielectric area than the cytosol, so the charge can be felt more strongly in the membrane at a greater distance. Second, MLP-1 is anchored by the cytoskeleton in cholesterol-containing lipid domains [48]. MLP-1 binding of PIP_2_ explains why PIP_2_ can also be found in these negatively charged lipids [59,60]. 

Second, unlike ligand binding electrostatic binding is not stoichiometric. The positive charge concentration of MLP-1 can associate with three or more PIP_2_. Discussing a specific binding constant is inappropriate, but the apparent binding constant is related to the depth of the energy well caused by MLP’s positive charge domain. This is the energy necessary to move one PIP_2_ from close to MLP-1 to free diffusion in the membrane lipid. Nonetheless, the PIP_2_ binding constant to MLP-1 has been variously reported from 10 to 30 μM. If we choose a dissociation constant of 15 μM, the MLP-1 energy well would be between 6 and 6.5 Kcal/mol. In order for the PIP_2_ associated with MLP-1 to activate ENaC, then PIP_2_ must gain enough energy to climb out MLP-1’s potential well and rapidly associate with ENaC. If the association of PIP_2_ causes ENaC to open, then the rate at which PIP_2_ leaves MLP-1 should be related to the opening rate of ENaC. We showed in Figure 10 that altering the charge structure of ENaC’s PIP_2_ binding domains altered channel open probability in a way predicted by an increased ability to bind PIP_2_.

These results describe an entirely different mechanism than ligand-receptor binding for delivering PIP_2_, a known signaling molecule, to proteins whose activity it modifies. The results will apply to other anionic lipids, e.g., phosphatidic acid and PIP_3_. Electrostatic interactions will require conceptualizing lipid-protein interactions in a different way than traditional receptor-ligand interactions. 

The binding of ENaC to the MH2 domain of MLP-1 means that ENaC and MLP-1 are close enough together to share a common charge cloud in which PIP_2_ moves easily between them. The charged domain of MLP-1 has a linear structure which, due to the strongly hydrophobic phenylalanines, resides in the membrane at the level of phospholipid head groups with the positive charge residues near the surface of the membrane [20]. This likely leads to an extended structure of the β and γ N-terminal ENaC domains along the inner surface of the membrane with the positively charged residues associating with PIP_2_ in the electrostatic cloud surrounding MLP-1 and ENaC (see graphical abstract). Although we described the rate of PIP_2_ *leaving* β or γ ENaC, a more realistic picture might be the N-terminal tails *leaving* PIP_2_ and moving from association with the membrane and MLP-1 to the cytosol, which would coincide with channel opening and closing. 

Phosphatidyl inositol 4,5-bisphosphate consists of an inositol head group and a diacylglycerol group. The acyl groups can vary, but typically one group is a stearyl group, and the other is an arachidonoyl group. This form of PIP_2_ has a low but finite solubility in saline. One of our first experiments examined the effect of PIP_2_ on ENaC open probability in excised patches from H441 cells. In this paper, we show that the activation is dose-dependent (Figure 3). When we fit the dose-response curve to the Hill equation (Equation (1) above), we found that half activating dose of PIP_2_, K_0.5_, was 22.5 ± 0.664 with a P_max_ of 0.41 ± 0.16. The K_0.5_ is consistent with the estimated concentration of 5–30 μM PIP_2_ in the apical membranes of epithelial cells. The Hill coefficient was 2.5 ± 0.55. The coefficient is interesting since it implies that it requires at least two PIP_2_ molecules per ENaC molecule. This is consistent with the multiple PIP_2_ binding sites in the N-termini of β and γ ENaC. 

The main effect of adding endogenous PIP_2_ to the cytosolic surface is to reduce the mean closed time; the reciprocal of the mean closed time is the opening rate which increases with increasing PIP_2_. The implication is that when endogenously applied, PIP_2_ binds to one or more of the binding sites on ENaC subunits that the ENaC opens and that as the PIP_2_ concentration increases, the channel opens more frequently. This observation is consistent with a first-order reaction, the rate of which depends upon the concentration of PIP_2_ and in which binding of one PIP_2_ binding to ENaC is sufficient to open the channel.

However, if PIP_2_ binding were a simple a simple pseudo-first-order reaction going from closed to open (Equation (4)), then the opening rate would depend on PIP_2_ concentration, as seen in Figure 2, and the closing rate (the reciprocal of the open time) would be a constant.

(4) $$closed\begin{array}{c} \left[PIP_{2}\right]k_{1}\\ ⇄\\ k_{-1} \end{array}open\;\;\;\;$$

An examination of the single channel records in Figure 3 shows that this is clearly not the case: as PIP_2_ increases, mean open time increases (closing rate decreases). This property of the mean open time was also observed by Pochynyuk [14]. This implies that the kinetic scheme must be more complicated than that shown in Equation (4). In fact, the Hill coefficient suggests that there must be multiple open states. Although there are many kinetic schemes that would produce the observed increase in open time, the simplest is probably

(5) $$closed\begin{array}{c} \left[PIP_{2}\right]k_{1}\\ ⇄\\ k_{-1} \end{array}open_{1}\;\;\begin{array}{c} \left[PIP_{2}\right]k_{2}\\ ⇄\\ k_{-2} \end{array}open_{2}\;\;\;\;$$

In this model, the binding of the first PIP_2_ opens the channel and the binding of the second stabilizes the channel in an open state. Both forward reactions depend upon PIP_2_ concentration. As PIP_2_ concentration increases, the rate of opening increases, but the rate of moving to the second open state also increases. The second open state can return to the first open state at a fixed rate (*k_−_*_2_), but after entering *OPEN_1_*, the channel can either close at a fixed rate (*k*_−1_) or the channel can re-enter the second open state with a rate that depends upon PIP_2_ concentration ([PIP_2_]k_1_). Therefore, as the PIP_2_ concentration increases, the channel resides in the *OPEN_2_* state more often, i.e., it remains open longer, as we observe.

**Figure 11:**
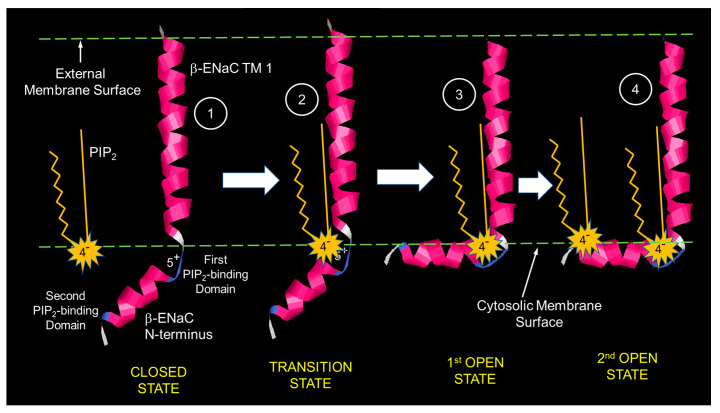
Schematic of PIP_2_ activation of ENaC. In 1, PIP2 and the N-terminus of b-ENaC are separated, and the cytosolic domain of b-ENaC is free; in 2, PIP_2_ is attracted to the positive charge of b-ENaC’s PIP_2_ binding domain; in 3, b-ENaC’s N-terminus folds to be in close association with PIP_2_′s head group and the inner surface of the membrane. The change in conformation also gates ENaC open transiently. In 4, the second PIP_2_ binding domain associates with a second PIP_2,_ which stabilizes ENaC, leading to the second open state. The channel may make many transitions back and forth between the two open states.

Our physical interpretation of our results is that the N-terminal cytosolic domains of the ENaC subunits can move freely in the cytosol and when free the channel is closed, but the electrostatic fields of PIP_2_ at the inner surface of the membrane biases the movement of the N-termini so that they the N-termini move toward the membrane surface; the binding site closest to the membrane (the binding site closest to the membrane-spanning domain) will be subject to the largest electrostatic force and will be first to associate with membrane PIP_2_. This initial electrostatic interaction is the transition to the first open state. With the N-terminus now constrained, the possibility of PIP_2_ binding to a second binding site is increased and dependent on the available PIP_2_. If this second binding occurs, the channel can move into the second open state, where its residency will increase the mean open time and the channel open probability. A schematic diagram of PIP_2_ activation is shown in Figure 11. 

However, we were perplexed by the cell properties since we had expected that a reduction in MLP-1 should produce an increase in the closed time (longer closures because less PIP_2_ was available to open ENaC), but instead, the open time is reduced. This implies a destabilization of ENaC in the absence of MLP-1. ENaC can no longer associate with MLP-1, but the results are as if ENaC is no longer tethered to the cytoskeleton and can only find ENaC as the result of a random walk. The implication appears to be that in the knockout cells, PIP_2_ is distributed uniformly in the membrane as opposed to being concentrated by MLP-1’s effector domain. This situation would be comparable to MLP-1 being disassociated with the membrane [1,9] or to an excised patch with no exogenous PIP_2_ on the cytosolic surface. The mean open time in the knockout cells is 41.1 ± 23.4 ms (mean ± s.d.), the mean open time of cells expressing an MLP-1 mutant that cannot associate with the apical membrane [1] is 81.9 ± 71.7 ms (mean ± s.d.), and the mean open time of an excised patch with no cytosolic PIP_2_ is 93.2 ± 43.6 ms (mean ± s.d.). Others have also described a reduction in mean open time associated with a reduction in PIP_2_; Pochynyuk and coworkers [61] showed that when purinergic receptor binding activated phospholipase C, which reduces PIP_2_ by degradation, the mean open time fell from 540 ± 45 to 129 ± 12 ms. 

We speculate that under conditions when PIP2 concentration is low (excised patches with no PIP2 or PIP2 degradation by PLC) or PIP2 is widely distributed in the membrane (MLP-1 knockout), ENaC (in Figure 11) moves only to the first open state, but rarely, if ever, because of the low density of PIP2 moves all the way to the stabilized second open state. This leads to frequent brief openings but few, if any, long openings. 

## 5. Conclusions

For years we have known that PIP_2_ was important for ENaC activity. In this paper, we describe a mechanism for PIP_2_ activation of ENaC. We have shown by several different experiments that the ENaC-PIP_2_ interaction is electrostatic, but the interaction is mediated by the membrane-associated protein, MLP-1, that locally concentrates PIP_2_ in the vicinity of ENaC to increase ENaC activity. 

The overall significance of this work is two-fold: first, it proposes a new paradigm for the regulation of ENaC involving ENaC’s presence in PIP_2_-rich lipid domains and the role and regulation of the chaperone, MLP-1, in controlling ENaC activity; and second, it will allow an understanding of the regulation of ENaC open probability and the role of this regulation in ENaC function. Defects in any component of this system could lead to serious pathologies of sodium transport and consequent abnormalities in blood pressure and fluid and electrolyte balance. 

## Data Availability

All data is provided in full in the results section of this paper.

## Supplementary Materials

The following supporting information can be downloaded at: https://www.mdpi.com/article/10.3390/biology11121694/s1.
