# Supplementary material for: PIP2 Interacts Electrostatically with MARCKS-like Protein-1 and ENaC in Renal Epithelial Cells
Source: Biology (Basel). 2022 Nov 24;11(12):1694. doi: 10.3390/biology11121694 (PMC9774185; doi:10.3390/biology11121694)
Supplement: Supplementary file 1 [file biology-11-01694-s001.zip › biology-1849136-supplementary.pdf]

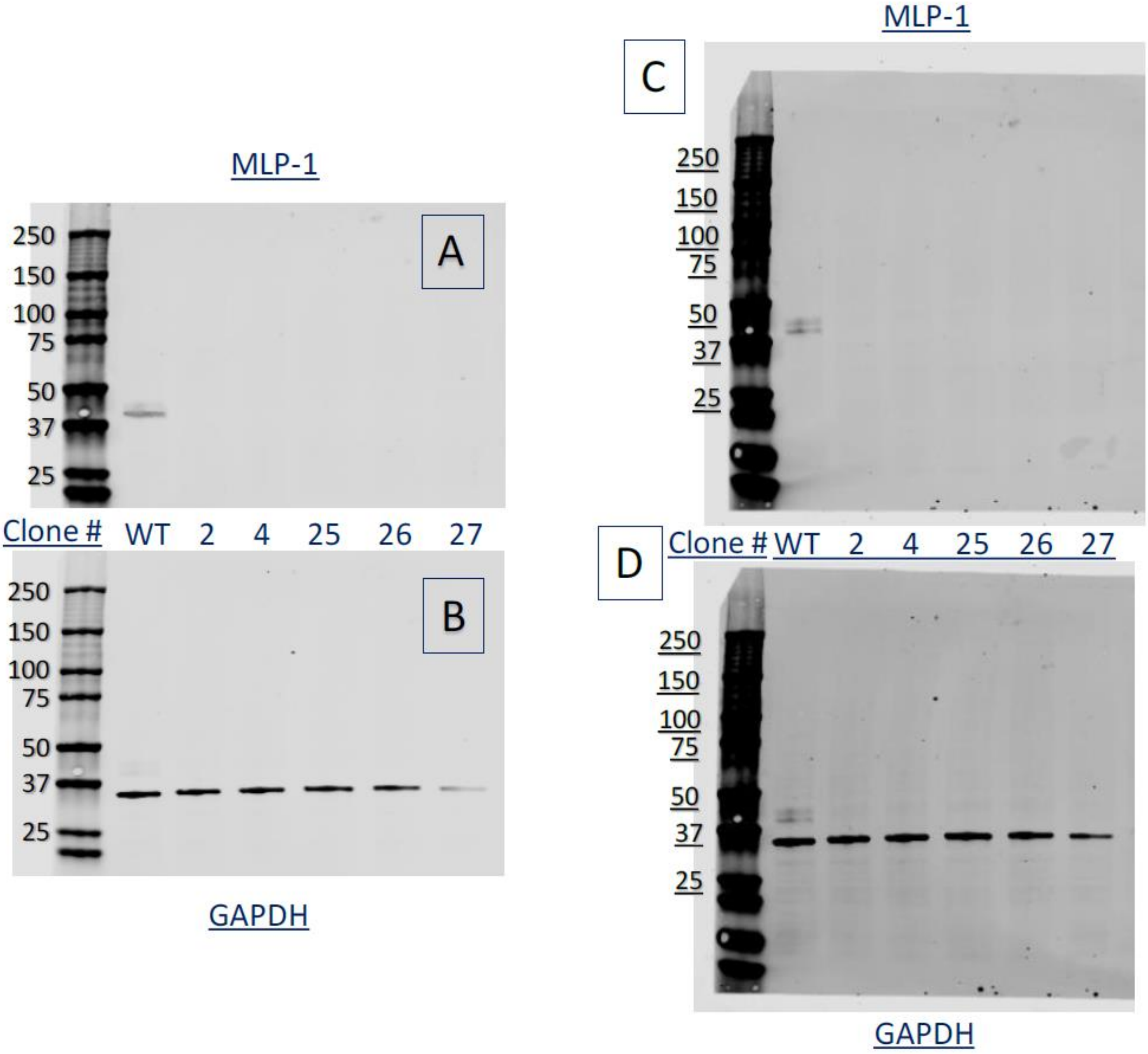

Supplement Figure S1. ENaC activity is greatly reduced in mpkCCD cells in which MLP-1 is knocked out. Western blots of wild type and 5 KO clones are shown in A blotted for MLP-1 or GAPDH (in B). MLP-1 is below detection limits in the clonal cells. 12µg total protein was loaded in each lane. On the right are images of the complete blots with C treated with anti-C MLP 1:2000 and D anti-C MLP + anti GAPDH 1:8000. Image D: anti-C MLP + anti GAPDH 1:8000.

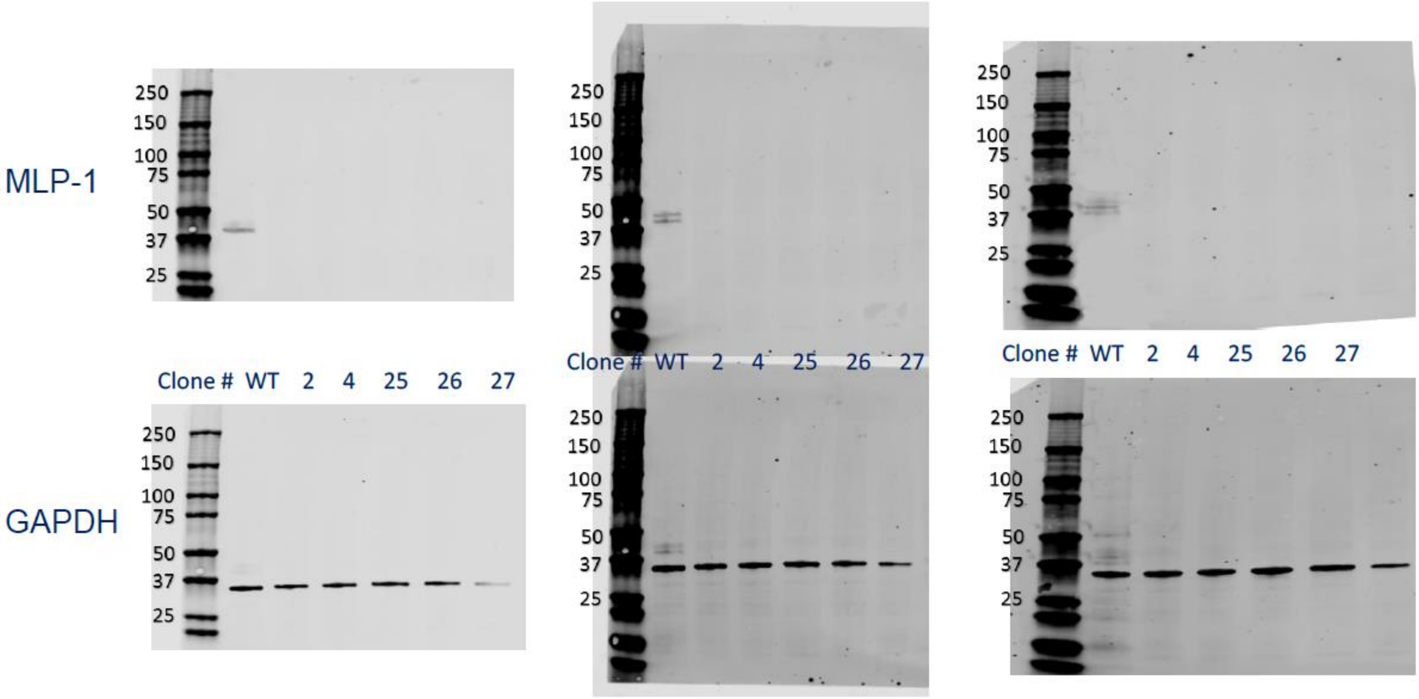

Supplement Figure S2. ENaC activity is greatly reduced in mpkCCD cells in which MLP-1 is knocked out. MLP-1 was knocked down 30-fold or more (by Q PCR) using Crispr/Cas9 in several clones. This figure shows 3 Western blots of wild type and 5 KO clones. Upper panels are blotted for MLP-1 (with antibodies described in methods). Wild type (in lane next to MW markers) show MLP-1; in all other lanes for KO clones, MLP-1 is below the level of detection. The lower panels are the same gels probed for GAPDH as a measure of protein loading (12 µg).

Antibodies in this paper. The following primary antibodies: C-terminal MLP (1:5000, Abcam Cat# ab184546), N-terminal MLP (1:1000, GeneTex Cat# GTX56062), flag (1:2000, Sigma-Aldrich Cat# F1804, RRID:AB_262044), mcherry (1:5000, OriGene Cat# TA150126), V5 (1:5000, Thermo Fisher Scientific Cat# R960-25, RRID:AB_2556564), α-ENaC (1:2000, StressMarq Biosciences Cat# SPC-403, RRID:AB_10640131), β-ENaC (1:2000, StressMarq Biosciences Cat# SPC-404, RRID:AB_10644173), and γ-ENaC (1:1000, StressMarq Biosciences Cat# SPC-405D-DY405, RRID:AB_2825485). The membranes were also probed for beta-actin antibody (Cell Signaling Technology Cat# 3700, RRID:AB_2242334) or GAPDH antibody (Cell Signaling Technology Cat# 97166, RRID:AB_2756824) at a dilution of 1:3000 for 2 hours at room temperature as a loading control. The membranes were washed three times with 1×TBST for 5 min intervals and then incubated with appropriate species-specific IR Dye secondary antibodies (LI-COR Biosciences Cat# 925-32211, RRID:AB_2651127, LI-COR Biosciences Cat# 925-68071, RRID:AB_2721181, LI-COR Biosciences Cat# 926-68072, RRID:AB_10953628, LI-COR Biosciences Cat# 926-68074, RRID:AB_10956736) at a dilution of 1:10,000.
